# Patient- and provider-related factors in the success of multidrug-resistant tuberculosis treatment in Colombia^*^

**DOI:** 10.26633/RPSP.2021.74

**Published:** 2021-06-21

**Authors:** Gloria Mercedes Puerto Castro, Fernando Nicolás Montes Zuluaga, Jacqueline Elizabeth Alcalde-Rabanal, Freddy Pérez

**Affiliations:** 1 Colombia National Network for Tuberculosis Research Innovation and Knowledge Management, National Institute of Health Bogotá Colombia Colombia National Network for Tuberculosis Research Innovation and Knowledge Management, National Institute of Health, Bogotá, Colombia.; 2 Medellín Municipal Secretariat of Health Medellín Colombia Medellín Municipal Secretariat of Health, Medellín, Colombia.; 3 Mexico National Institute of Public Health, Health Systems Research Center, Cuernavaca Morelos Mexico Mexico National Institute of Public Health, Health Systems Research Center, Cuernavaca, Morelos, Mexico.; 4 Department of Communicable Diseases and Environmental Determinants of Health, Pan American Health Organization Washington, DC USA Department of Communicable Diseases and Environmental Determinants of Health, Pan American Health Organization, Washington, DC, USA.

**Keywords:** Tuberculosis, multidrug bacterial resistance, health personnel, health human resources training, Colombia, Tuberculosis, farmacorresistencia bacteriana multiple, personal de salud, capacitacion de recursos humanos en salud, Colombia, Tuberculose, farmacorresistência bacteriana múltipla, pessoal de saúde, capacitação de recursos humanos em saúde, Colômbia

## Abstract

**Objective.:**

To identify patient- and provider-related factors associated with the success of multidrug-resistant tuberculosis (MDR-TB) treatment in the six municipalities of Colombia with the highest number of MDR-TB cases.

**Methods.:**

Bivariate and multivariate logistic regressions were used to analyze the association between treatment success (cure or treatment completion) and characteristics of the patients and physicians, nursing professionals, and psychologists involved in their treatment. The importance of knowledge in the management of MDR-TB cases was explored through focus groups with these providers.

**Results.:**

Of 128 cases of TB-MDR, 63 (49.2%) experienced treatment success. Only 52.9% of the physicians and nursing professionals had satisfactory knowledge about MDR-TB. Logistic regression showed that being HIV negative, being affiliated with the contributory health insurance scheme, being cared for by a male physician, and being cared for by nursing professionals with sufficient knowledge were associated with a successful treatment outcome (*p* ≤ 0.05). Qualitative analysis showed the need for in-depth, systematic training of health personnel who care for patients with MDR-TB.

**Conclusions.:**

Some characteristics of patients and healthcare providers influence treatment success in MDR-TB cases. Physicians’ and nurses’ knowledge about MDR-TB must be improved, and follow-up of MDR-TB patients who are living with HIV and of those affiliated with the subsidized health insurance scheme in Colombia must be strengthened, as these patients have a lower likelihood of a successful treatment outcome.

## RESUMEN

**Objetivo.:**

Identificar los factores asociados con el éxito del tratamiento de tuberculosis multidrogorresistente (TB-MDR) relacionados con los pacientes y el personal sanitario en seis municipios de Colombia con mayor número de casos.

**Métodos.:**

Mediante regresiones logísticas bifactorial y multifactorial se analizó la asociación entre el tratamiento exitoso (curación o cumplimiento del tratamiento) y las características de los pacientes, y de los médicos, profesionales de enfermería y psicólogos vinculados al tratamiento. Se exploró la importancia del conocimiento en el manejo de los casos de TB-MDR mediante grupos focales con esos profesionales.

**Resultados.:**

De los 128 casos con TB-MDR, 63 (49,2%) tuvieron un tratamiento exitoso. Solo 52,9% de los médicos y profesionales de enfermería tenía conocimientos satisfactorios sobre TB-MDR. La regresión logística mostró que ser negativo al VIH, estar afiliado al régimen de aseguramiento de salud contributivo, estar atendido por un médico del sexo masculino y por profesionales de enfermería con conocimientos suficientes se asociaron con un desenlace exitoso del tratamiento (p ≤ 0,05). El análisis cualitativo mostró la necesidad de profundizar y sistematizar la capacitación del personal sanitario que atiende los casos de TB-MDR.

**Conclusiones.:**

En el éxito del tratamiento de los casos de TB-MDR influyen algunas características de los pacientes y el personal sanitario. Se requiere fortalecer los conocimientos sobre TB-MDR de médicos y enfermeros, y reforzar el seguimiento de los pacientes con TB-MDR positivos al VIH y de los que pertenecen al régimen subsidiado, dada su menor probabilidad de éxito al tratamiento.

## RESUMO

**Objetivo.:**

Identificar os fatores associados ao êxito do tratamento da tuberculose multirresistente (TBMR) relacionados ao paciente e à equipe de saúde nos seis municípios da Colômbia com o maior número de casos.

**Métodos.:**

Mediante regressão logística bifatorial e multifatorial, analisou-se a associação entre o êxito do tratamento (cura ou completude do tratamento) e as características dos pacientes e dos médicos, profissionais de enfermagem e psicólogos envolvidos neste. Explorou-se a importância do conhecimento no manejo de casos de TBMR mediante grupos focais com os mesmos profissionais.

**Resultados.:**

Dos 128 casos de TBMR, 63 (49.2%) lograram êxito no tratamento. Somente 52.9% dos médicos e profissionais de enfermagem tinham conhecimentos satisfatórios sobre TBMR. A regressão logística demonstrou que soronegatividade para o HIV, cobertura pelo sistema de saúde sob o regime de contribuinte, atendimento por um médico do sexo masculino e atendimento por profissionais de enfermagem com conhecimento suficiente foram fatores associados ao êxito do tratamento (*p* ≤ 0,05). A análise qualitativa demonstrou necessidade de aprofundar e sistematizar a capacitação do pessoal de saúde que atende casos de TBMR.

**Conclusões.:**

Algumas características do paciente e da equipe de saúde influenciam no êxito do tratamento de casos de TBMR. É preciso fortalecer os conhecimentos dos médicos e profissionais de enfermagem sobre a TBMR e reforçar o seguimento dos pacientes com TBMR que vivem com HIV e os filiados ao sistema de saúde colombiano pelo regime subsidiado, os quais têm menor probabilidade de êxito do tratamento.

Drug-resistant tuberculosis (TB) is a public health threat in Colombia and around the world. The World Health Organization (WHO) estimated that 500 000 new cases of TB were resistant to rifampicin in 2019; of these, 390 000 were multidrug-resistant (MDR-TB), i.e., resistant both to rifampicin and isoniazid, the two first-line antibiotics used to treat the disease (1). The Region of the Americas ranks sixth in the world in number of MDR-TB cases, with an estimated incidence of one case per 100 000 population in 2019 (1). In Colombia, the incidence of MDR-TB that year was 1.2 cases per 100 000, making it the country with the seventh largest number of MDR-TB cases in the Region (2). The departments with the highest cumulative incidence, together accounting for more than 50% of the MDR-TB cases in Colombia, are Valle del Cauca, Antioquia, Risaralda, Atlántico, and Bogotá. The cities most affected are Medellín, Cali, Pereira, Barraquilla, and Bogotá (3).

To tackle this public health challenge, the Ministry of Health and Social Protection created the National Tuberculosis Control and Prevention Program (PNPCT, by its Spanish acronym), which has proposed mandatory programmatic guidelines to be followed by the country’s health service institutions (HSIs). Success in treating MDR-TB cases in Colombia increased from 38% in 2014 to 44% in 2017 (4, 5). However, despite this progress, the country is still far from meeting the proposed WHO target of successfully treating 75% of MDR-TB cases by 2020 and 90% by 2025 (6). According to several reports in the literature, treatment failure can be attributed to a number of different factors, including male gender; over 50 years of age; presence of comorbidities, including human immunodeficiency virus (HIV) infection (7); alcohol use; social factors, such as stigma and discrimination (8); and financial limitations (9, 10). On the other hand, a positive improvement in treatment has been seen when health personnel have satisfactory knowledge about the management of TB cases (10, 11). Isara and Akpodiete (12) found that insufficient knowledge about TB on the part of health providers correlated with poorer treatment outcomes. It has been reported that when these professionals have satisfactory knowledge, good practices are followed in treating TB patients (13).

While it was found in Bogotá that many professionals caring for TB patients did not have enough knowledge about the disease, including its prevention, diagnosis, treatment, and follow-up (14, 15), there are no recent studies in Colombia that show which factors are associated with treatment success in patients with MDR-TB. The objective of the present study was to identify patient- and provider-related factors correlated with MDR-TB treatment success in the six municipalities of Colombia with the highest numbers of these cases.

## MATERIALS AND METHODS

An implementation study was conducted using a cross-sectional observational design with a concurrent mixed methods approach (16). The study was conducted in HSIs in the municipalities of Medellín and Bello (Department of Antioquia), Pereira and Dosquebradas (Department de Risaralda), and Cali and Buenaventura (Department of Valle del Cauca), which diagnosed the largest number of MDR-TB cases between January 2016 and June 2018.

The quantitative component explored the individual characteristics of MDR-TB patients and the health professionals responsible for programmatic management of TB cases (physicians, nursing professionals, and psychologists), as well as their participation in training events over the past year. In addition, the knowledge of physicians and nursing professionals regarding the program management of MDR-TB cases was assessed. For this purpose, a questionnaire was developed consisting of 10 multiple-choice questions covering the definition of MDR-TB, treatment regimens, phases for new and previously treated patients, frequency of follow-up by the general physician and nursing personnel, and bacteriological monitoring. The relevance and clarity of the questions were previously validated in an HSI not included in the study. Next, three physicians specialized in the management of MDR-TB reviewed and evaluated the questionnaire. They decided that the knowledge was sufficient when 70% of the questions were answered correctly (11, 13).

The characteristics of the patients and health providers were analyzed descriptively based on their occupational category, then bivariate analysis was used to estimate the correlation between these individual characteristics and treatment success. Treatment was considered successful when the patient was cured or completed treatment, and unsuccessful if the patient died or interrupted follow-up for more than 30 days (17).

For the multivariate regression analysis (18), the dependent variable was treatment success and the predictive variables were the individual characteristics of the patients (Table 1) and the health providers (Table 2).

The variables that were statistically significant (*p* ≤ 0.05) in the bivariate analysis were then used as explanatory variables to develop step-by-step logistic regression models (18). Three models were tested: model A assumed that the predictors were the individual patient variables; model B assumed that they were the individual characteristics of the health providers; and model C included the individual variables of both the patients and the providers. Adjustment of the logistic models was confirmed post hoc using the Hosmer-Lemeshow test. The statistical package used was STATA 14^®^ (StataCorp, College Station, Texas, USA).

In the qualitative component, nine focus groups were created with personnel responsible for the TB program in the HSIs: three with physicians, three with nursing professionals, and three with psychologists. The invitation to participate in the focus groups was open to all professionals involved in the TB program. The health professionals were asked for their impressions of the training they received in the departmental or municipal health secretariats and the extent to which it had influenced their knowledge about the programmatic management of MDR-TB cases in terms of the frequency, intensity, and relevance of the training, as well as continuity and workload. The focus group discussions were recorded and transcribed in Microsoft Word. The coding and category content analysis were done using the Atlas.ti 8.0 workbench^®^ (19). Finally, the methods were integrated for comparison of the results (16). The questions requiring narrative answers were designed to seek explanations of the results.

The study was authorized by the Ethics and Research Methodology Committee of the National Institute of Health in Bogotá, Colombia, and also the Ethics Committee of the Pan American Health Organization. The participants’ informed consent was obtained in writing. No names were collected. The instruments used processing codes, so it would be impossible to identify the participants.

## RESULTS

During the course of the study, a total of 128 patients with MDR-TB received care in 51 HSIs, and of this number, 63 (49.2%) were successfully treated. The variables HIV—negative (*p* = 0.02) and coverage under the contributory health insurance scheme (*p* = 0.001) correlated with treatment success. However, there was no significant correlation between treatment success and the other variables: gender, age, municipality of residence, type of TB (pulmonary or extrapulmonary), or status upon entering the program (new or previously treated case) (Table 1).

### Quantitative analysis

All the HSIs had a physician and a nursing professional for patient care, and 37 (72.5%) of them also had a psychologist. All the HSI physicians and nursing professionals involved in the TB program (*n* = 102) responded the questionnaire. Of the 139 professionals providing care, 62.6% were female and the 31-to-50 age group was the largest (58.3%); 46.8% had between one and five years’ experience in the TB program and 25.2% had been in the program for more than five years. Of the total respondents, 26 general practitioners (51.0%), 11 nursing professionals (21.6%), and 27 psychologists (73.0%) had not received any specific training in MDR-TB during the past year. Only 27 physicians (52.9%) and 27 nursing professionals (52.9%) proved to have sufficient knowledge of MDR-TB case management according to the country’s current guidelines (Table 2). The percentages of correct responses to each question are shown in Table 3.

**TABLE 1.** Clinical manifestations, demographic characteristics, and outcomes in the treatment of multidrug-resistant tuberculosis in six municipalities of Colombia, 2016-2018

**Variable**	**Successful treatment** **(n = 63)**		**Unsuccessful treatment** **(n = 65)**		**Total** **(n = 128)**	***p*^**a**^**
	**n**	%	**n**	%		
Sex						
Male	40	63.5	49	75.4	89	0.143
Female	23	36.5	16	24.6	39	0.337
Age (years)						
20 or under	5	7.9	6	9.2	11	
21 to 39	26	41.3	30	46.2	56	
40 to 60	27	42.9	19	29.2	46	
Over 60	5	7.9	10	15.4	15	
Residence (municipality)						
Medellín	31	49.2	26	40.0	57	0.399
Cali	14	22.2	21	32.3	35	
Buenaventura	7	11.1	9	13.8	16	
Pereira	6	9.5	7	10.8	13	
Dosquebradas	2	3.2	2	3.1	4	
Bello	3	4.8	0	0	3	
Type of tuberculosis						
Pulmonary	59	93.7	62	95.4	121	0.666
Extrapulmonary	4	6.3	3	4.6	7	
HIV status						
Negative	56	88.9	45	69.2	101	0.020
Positive	6	9.5	16	24.6	22	
No data	1	1.6	4	6.2	5	
Status upon entering the program						
New case	35	55.6	35	53.8	70	0.845
Previously treated case	28	44.4	30	46.2	58	
Health insurance system						
Subsidized^b^	30	47.6	49	75.4	79	0.001
Contributory^c^	33	52.4	16	24.6	49	

***Source:*** Own preparation.

HIV, human immunodeficiency virus.

a Chi-square test level of significance: *p* ≤ 0.05.

b Insurance for health services covered by the State.

c Insurance for health services covered by the user.

Table 4 presents the logistic regression results for the three models analyzed. In model A, which considered the patient explanatory variables, only two variables correlated statistically with treatment success: affiliation with the contributory health insurance scheme and HIV-negative status (Nagelkerke *R*^2^ = 0.1435; *χ*^2^ = 25.47; *p* < 0.0025). In model B, based on the provider explanatory variables, the following were statistically significant: sufficient knowledge of MDR-TB (for nursing professionals) and male gender (for physicians) (Nagelkerke *R*^2^ = 0.1876; *χ*^2^ = 18.59; *p* < 0.0023). In model C, which combined both the patient and provider explanatory variables, a statistically significant correlation was found between treatment success and affiliation with the contributory health insurance scheme, HIV-negative status, and treatment by a male physician (Nagelkerke *R*^2^ = 0.2948; *χ*^2^ = 32.56; *p* < 0.001).

### Qualitative analysis

Each focus group consisted of six to eight professionals. Analysis of their comments revealed situations that hinder the acquisition of knowledge during PNPCT training activities or those offered by the departmental and municipal health secretariats. According to the participants, the frequency of attendance and intensity of the training declined when the providers had a heavy workload. High staff turnover was also an issue in the institutions where they work.

**TABLE 2.** Characteristics of health service providers, by occupational category, in health service institutions in the municipalities studied. Colombia, 2019

Variable	Physicians (n = 51)		Nursing professionals (n = 51)		Psychologists (n = 37)		p^a^
	n	%	n	%	N	%	
Gender							
Male	23	45.1	0	0	29	78.4	< 0.001
Female	28	54.9	51	100.0	8	21.6	
Age (years)							
30 or under	17	33.3	13	25.5	10	27.0	0.712
31 to 50	26	51.0	33	64.7	22	59.5	
Over 50	8	15.7	5	9.8	5	13.5	
Years of experience in the TB program							
Less than 1	18	35.3	12	23.5	9	24.3	< 0.001
1 to 5	22	43.1	17	33.3	26	70.3	
More than 5	11	21.6	22	43.1	2	5.4	
Years since graduation							
5 or under	23	45.1	18	35.3	11	29.7	0.053
6 to 10	9	17.6	13	25.5	17	45.9	
More than 10	19	37.3	20	39.2	9	24.3	
Training received							
Yes	25	49.0	40	78.4	10	27.0	< 0.001
No	26	51.0	11	21.6	27	73.0	
Knowledge of MDR-TB							
Sufficient	27	52.9	27	52.9	NA	NA	0.834
Insufficient	24	47.1	24	47.1	NA	NA	

***Source:*** Own preparation.

TB, tuberculosis; MDR-TB, multidrug-resistant tuberculosis; NA, not applicable.

a Chi-square test level of significance: *p* ≤ 0.05.

**TABLE 3.** Proportion of correct responses by physicians and nursing professionals in questionnaire on knowledge of programmatic management of multidrug-resistant tuberculosis (MDR-TB)

**Question**	**Physicians** **(*n* = 51)**		**Nursing professionals (n = 51)**		**Total for both**	***p*^a^**
	*n*	%	*n*	%		
1. What is MDR-TB?	45	88.2	39	76.5	84	0.194
2. What is the treatment regimen for a new patient with MDR—TB?	34	66.7	34	66.7	68	0.833
3. What is the treatment regimen for a previously treated MDR-TB patient?	37	72.5	31	60.8	68	0.293
4. What are the phases of treatment?	36	70.6	37	72.5	73	1.000
5. What is the indicator for moving from the intensive phase to follow-up?	38	74.5	37	72.5	75	1.000
6. Where can I find the drug dosage guidelines?	22	43.1	19	37.3	41	0.686
7. How often should patients be checked by the specialized physician?	26	51.0	22	43.1	48	0.551
8. How often should patients be checked by the general practitioner?	45	88.2	37	72.5	82	0.080
9. How often should patients be checked by the head nurse?	10	19.6	22	43.1	32	0.018
10. How often should cultures be taken during follow-up?	37	72.5	41	80.4	78	0.483

***Source:*** Own preparation.

a Chi-square test level of significance: *p* ≤ 0.05.

*Previously, the TB Program Coordinator met with the physicians and nurses every month to bring us up to date on all the latest guidelines and treatments, and also to motivate us. The meetings were very interesting. They made our work better. —*GF-BN-PE

*The head nurses are responsible for programming the training, but they are a transient population: they come and they go; their contract ends and new people arrive. These administrative hurdles tend to interrupt the continuity of the program. —*GF-PE-MG

**TABLE 4.** Logistic regression models designed to identify correlations with successful treatment of multidrug-resistant tuberculosis (TB) in six municipalities, Colombia, 2016-2018

**Variable**	**OR^a^**	**CI 95%^b^**	***p*^a^**
A. Patient explanatory variables (*R*^2^= 0.143)			
Gender	0.618	0.252-15.14	0.293
Health insurance coverage	3.985	1.698-9.354	0.001
HIV-negative status	0.357	0.149-0.854	0.021
Previous TB treatment	1.547	0.665-3.598	0.311
Additional drug resistance	1.005	0.432-2.334	0.99
Comorbidities	1.158	0.510-2.631	0.725
Age (years)			
21 to 39	1.103	0.263-4.617	0.892
40 to 60	2.643	0.564-12.377	0.217
60+	0.480	0.080-2.871	0.421
B. Provider explanatory variables (*R*^2^ = 0.187)			
Gender of the physician	5.627	1.738-18.214	0.004
Presence of a trained psychologist	1.742	0.514-5.905	0.372
Sufficient knowledge of the physician	0.244	0.042-1.404	0.114
Sufficient knowledge of the nursing professional	12.855	1.849-89.359	0.010
Nursing professional’s years of experience with TB	0.998	0.876-1.136	0.977
C. Patient and provider explanatory variables (*R*^2^ = 0.2948)			
Affiliation with the contributory system	5.442	1.589-18.632	0.007
HIV-negative status	0.251	0.061-1.031	0.055
Complete interdisciplinary group	0.478	0.097-2.354	0.364
Gender of the physician	3.665	0.967-13.881	0.056
Nursing professional’s years of experience with TB			
2 to 5	0.982	0.174-5.531	0.984
5+	3.416	0.709-16.443	0.125
Presence of a trained psychologist	2.099	0.487-9.032	0.319

***Source:*** Own preparation.

a OR: odds ratios (*odds ratio*).

b CI 95%: 95% confidence interval.

c *p*: Wald test significance level *p* ≤ 0.05.

With regard to the relevance of the trainings, the informants reported that human resources, time, and space were insufficient and that the content was inadequate.

*The program made me aware that there is not enough training in how to deal with this disease. I’ve had training in in-facility care, certification, guidelines, and all that, but I would still like to hear more from an infectious disease specialist on how to address this disease—also, from the laboratory, on how to work with it, because, frankly, we didn’t learn that in our training. —*GF-EP-PE

The providers also mentioned the need to receive more in-depth knowledge about MDR-TB from specialized professionals, as well and more information on diagnostic and therapeutic techniques. They said that their time and opportunities available to take advantage of the training were limited by the fact they had so much work to do that they were forced to use their free time for training.

*It would be a good idea for the Ministry to offer some courses given by doctors who are experts in the technology, including basic training on multidrug resistance. If we keep looking at the situation like something from outer space, we are going to continue to have problems: We need to land. —GF-EP-ME*

*It seems me that the departmental or municipal government should be responsible. The training that’s offered is limited, and when they do schedule it and invite us to take it, we already have so much to do that there’s no way we can attend. —GF-EP-PS*

With regard to content, the providers requested basic training in MDR-TB, noting that TB is seen as an “unusual” problem and not part of the country’s epidemiological and public health reality in terms of infectious disease.

*We have the capacity and the technology to see all patients. Why isn’t this working? The problem is human resources. They never train us in TB. That’s the truth. No one receives it: not the doctors, not the nurses, not the psychologists, no one. —*GF-EP-PE

*We receive training in hypertension, diabetes. For example, all the outpatient doctors have received training on cardiovascular risk, but nothing on infectious risk. —*GF-EP-MG

The interviewees said that they received very little information about TB in their university courses and did not know the PNPCT existed or how it operates. They also noted that the HSIs allow little time for training and that the trend toward virtual training is detracting from its quality.

*For me—I graduated more than more than 15 years ago—the training program helps to fill in the gaps. It brings us up to date on the latest in public health and epidemiological surveillance. They give us international guidelines, but they don’t focus on our local context. —*GF-CL-MD

*There are many virtual courses, but they don’t give us the time to take them. We have to take them at night, as if it we were still on the job. The training should be done during working hours; extra time should be allowed for it. —*GF-ME-ME

## DISCUSSION

This is the first study to explore the relationship between patient and provider characteristics and the successful management of MDR-TB in Colombia––information that is useful in proposing actions to improve implementation of the national program, as well as outcomes in the management of this condition.

The patient characteristics associated with successful treatment could explain, in part, why WHO’s proposed goal of 75% cure was not met in 2020 (6). For the most part, the cases occurred in men between 40 and 60 years old. This age range corresponds to worldwide patterns of TB cases, which mainly occur in men (1). However, more than half the patients in our study were new cases—in other words, they had not been previously treated for TB. This result is inconsistent with national and global patterns, where the largest proportion of MDR-TB cases occur in previously treated patients, most of whom developed drug resistance because they failed to complete their treatment (1, 20). Our finding is significant because it reveals community transmission of the disease and failure of the programs to break the chain of MDR-TB transmission in the population (21, 22).

With regard to the provider characteristics, the study found that, at the time of our research, 27.5% of the HSIs lacked a complete multidisciplinary health team for comprehensive patient care. The teams consisted of only a general practitioner and a nurse; there was no psychologist. This situation, seen in both public and private HSIs, is concerning because in Colombia it is mandatory for the health team to include a psychology professional under the MDR-TB program management guidelines (17). Insurers should insist on the presence of a complete team in which the physician sees patients on a regular monthly schedule; nursing personnel provide patient guidance, clinical and bacteriological monitoring, and actions in the areas of promotion and prevention; and the psychologist provides emotional support at the start of treatment and in quarterly consultations (17). Emotional support is essential for several reasons: these patients face psychological and social problems within their families and in the community, as well as stigmatization and discrimination (8).

A high percentage of professionals had not received training in MDR-TB programmatic management in the last year: 73.0% of the psychologists, 51.0% of the physicians, and 21.6% of the nursing professionals. A similar situation was observed in Arequipa, Peru, where physicians and nurses who saw MDR-TB patients had not received training for three years or had never received it at all (23). Inadequate health provider training has also been reported in clinics in Brazil (24). In Colombia, each territorial entity is tasked with meeting the established training standards for these health providers in order to build optimal care capacity for patients with MDR-TB. Also, insurers are responsible for ongoing human resources training (17).

It was also found that a high percentage of the physicians and nursing professionals had insufficient knowledge of programmatic management of MDR-TB. Shortcomings were identified in such key areas as the definition of MDR-TB, treatment regimens, drug dosages, and frequency of medical and nursing follow-up. Similar situations been identified elsewhere: A systematic review of knowledge about MDR-TB treatment showed that physicians did not know the therapeutic regimens, drug dosages, or duration of treatment (25). In Mexico, a sizable majority of health professionals working in public institutions had insufficient knowledge about TB; only 18% demonstrated that they had adequate knowledge (26).

In the logistic regression models analyzed, model A showed a significant correlation between treatment success and affiliation with the contributory health insurance system and HIV-negative status. In Colombia, the health services (benefits plans) offered for MDR-TB care are the same under both the health insurance schemes, so there is no reason to expect differences between them in terms of health outcomes. However, more favorable outcomes were observed for patients under the contributory insurance scheme compared with those in the subsidized system (27). This difference may be related to the quality of the care offered (28) or to social determinants of health, since the patients insured under the contributory regime had more socioeconomic advantages than those in the subsidized system (27, 29). Another variable, HIV infection, is a comorbidity associated with the development of TB and a recognized risk factor for unsuccessful treatment in cases of MDR-TB (30, 31).

Model B showed that adequate knowledge on the part of nursing professionals correlated significantly with treatment success. This finding confirms the important role of human resources in treating patients with MDR-TB and reaffirms the WHO position that it is necessary to strengthen human resources for health (in terms of quantity, quality, and competencies) in order to respond to the challenges currently facing public health (32). It has also been pointed out that human resource limitations in the sector may make it more difficult to achieve the Sustainable Development Goals in low- and middle-income countries, particularly the targets for TB (33).

There was also a significant correlation between successful management MDR-TB cases and male physicians, which differs from the results of a study by Berthold et al., in which it was found that female doctors offered better quality care than men (34). That said, it has also been documented that male doctors are more likely to be hired than women, which might give them more experience in managing the disease (35). These two findings would support the conclusion that, at least in some situations, the physician’s gender correlates with successful treatment outcome.

When analyzing the results, it should be kept in mind that the study had a few limitations, including the different training received by health workers. Factors such as subject matter, number of days, frequency, and modality were not considered in the present research. Absence of these distinctions may have affected the representation of proficiency levels and correlations with treatment outcome. Another limitation was that patient variables such as socioeconomic and the educational levels were not included. Also, no attempt was made to rate the quality of care offered in the different health units.

In conclusion, based on the results obtained, it can be said that MDR-TB treatment success in the cities studied is influenced both by individual characteristics of the patient (health insurance affiliation and HIV infection) and certain characteristics of the health providers—in particular, nursing professionals’ knowledge of programmatic guidelines for the management of MDR-TB cases, as well as male physicians.

The following measures are recommended for improving the PNPCT’s operations and treatment outcomes for patients with MDR-TB: a) district and municipal health secretariats should ensure that HSI health personnel receive ongoing training in the programmatic management of MDR-TB cases; b) HSIs should ensure that nursing professionals providing patient care have sufficient knowledge about the programmatic management of MDR-TB, based on the guidelines issued by the Ministry of Health and Social Protection, and they should strive to maintain continuity of experienced personnel in the services; c) further attention should be given to the differences in treatment outcomes related to the health insurance scheme with which the patient is affiliated; d) the HSIs should closely monitor HIV-positive cases of MDR-TB with a view to ensuring treatment success; and e) the PNPCT should partner with institutions offering advanced education in the health professions to disseminate information about the program’s work and training in MDR-TB.

## Disclaimer.

Authors hold sole responsibility for the views expressed in the manuscript, which may not necessarily reflect the opinion or policy of the *RPSP/PAJPH* and/or the Pan American Health Organization.
